# Clay exfoliation and polymer/clay aerogels by supercritical carbon dioxide

**DOI:** 10.3389/fchem.2013.00028

**Published:** 2013-11-13

**Authors:** Simona Longo, Marco Mauro, Christophe Daniel, Maurizio Galimberti, Gaetano Guerra

**Affiliations:** ^1^Department of Chemistry and Biology and INSTM Research Units, Università degli Studi di SalernoFisciano, Italy; ^2^Department of Chemistry, Materials and Chemical Engineering, Politecnico di MilanoMilano, Italy; ^3^CNR, Istituto per lo Studio delle Macromolecole, National Research CouncilMilano, Italy

**Keywords:** cationic clays, organoclays, montmorillonite, syndiotactic polystyrene, nanoporous-crystalline δ form, monolithic aerogels

## Abstract

Supercritical carbon dioxide (scCO_2_) treatments of a montmorillonite (MMT) intercalated with ammonium cations bearing two long hydrocarbon tails (organo-modified MMT, OMMT) led to OMMT exfoliation, with loss of the long-range order in the packing of the hydrocarbon tails and maintenance of the long-range order in the clay layers. The intercalated and the derived exfoliated OMMT have been deeply characterized, mainly by X-ray diffraction analyses. Monolithic composite aerogels, with large amounts of both intercalated and exfoliated OMMT and including the nanoporous-crystalline δ form of syndiotactic polystyrene (s-PS), have been prepared, by scCO_2_ extractions of s-PS-based gels. Also for high OMMT content, the gel and aerogel preparation procedures occur without re-aggregation of the exfoliated clay, which is instead observed for other kinds of polymer processing. Aerogels with the exfoliated OMMT have more even dispersion of the clay layers, higher elastic modulus and larger surface area than aerogels with the intercalated OMMT. Extremely light materials with relevant transport properties could be prepared. Moreover, s-PS-based aerogels with exfoliated OMMT could be helpful for the handling of exfoliated clay minerals.

## Introduction

Over the last decades, clay polymer nanocomposites (CPN) (LeBaron et al., 1999; Alexandre and Dubois, 2000; Ray and Okamoto, 2003; Bergaya, 2008; Chen et al., 2008; Galimberti, 2011) have been steadily increasing their importance in the field of material science, as they substantially improve polymer properties such as mechanical reinforcement, impermeability, thermal stability (Paul and Robeson, 2008; Bergaya and Lagaly, 2013). Superior properties are achieved when individual clay layers or stacks of few layers are evenly distributed in the polymer matrix and polymer-clay interfaces are maximized (Kojima et al., 1993; Vaia et al., 1993; Manias et al., 2001).

CPN exhibiting exfoliated clays are difficult to attain, particularly in the case of non-polar polymers (Choi et al., 2004; Robello et al., 2004; Chou and Lin, 2005; Galimberti et al., 2007, 2009, 2010).

Many reports show that different processing techniques based on supercritical carbon dioxide (scCO_2_) constitute effective ways to increase dispersion and delamination in polymer/clay nanocomposites (Ma et al., 2007; Nguyen and Baird, 2007; Treece and Oberhauser, 2007; Samaniuk et al., 2009; Baker et al., 2011; Feng-hua et al., 2011; Chen et al., 2012). However, X-ray characterization of most samples show the presence of basal 00*l* reflections, clearly indicating that treatments with scCO_2_ are generally unsui to induce complete organoclay exfoliation (Ma et al., 2007; Nguyen and Baird, 2007; Treece and Oberhauser, 2007; Samaniuk et al., 2009; Thompson et al., 2009; Baker et al., 2011; Feng-hua et al., 2011; Chen et al., 2012).

Only some reports, from the Kannan's group, show that a complete disappearance of the 00*l* reflections (and hence a complete exfoliation) can be achieved by scCO_2_ treatments on pure organoclays, where alkali counterions have been exchanged with long-chain alkylammoniums (Horsch et al., 2006; Manitiu et al., 2008). However, as a consequence of preparation of polymer nanocomposites, the 00*l* reflections reappear with peak height and location essentially independent of the processing conditions (Manitiu et al., 2008).

scCO_2_ treatments are also effective to prepare monolithic aerogels, by drying of wet gels. Aerogels constitute a unique class of materials, characterized by a highly porous network being attractive for many applications such as thermal and acoustic insulation, capacitors or catalysis (Kistler, 1931; Schaefer and Keefer, 1986; Anderson et al., 2002; Malik et al., 2006; Wei et al., 2010; Kucheyev et al., 2012; Longo et al., 2013). In recent years, the scCO_2_ extraction of gels of suitable thermoplastic polymers, like syndiotactic polystyrene (s-PS) (De Rosa et al., 1997; Milano et al., 2001; Gowd et al., 2006; Rizzo et al., 2007; Petraccone et al., 2008) and poly(2,6-dimethyl-1,4-phenylene)oxide, (Daniel et al., 2011, 2013a, b; Galizia et al., 2012; Tarallo et al., 2012) has allowed the preparation of a special class of monolithic aerogels, (Daniel et al., 2005, 2008, 2009, 2012, 2013a, b; Wang and Jana, 2013) that present, beside disordered amorphous micropores (typical of all aerogels), identical nanopores of *nanoporous-crystalline* phases. Many studies show that nanoporous-crystalline polymer phases are able to absorb low molecular-mass molecules also when present in traces and are suitable for molecular separation (Manfredi et al., 1997; Musto et al., 2002; Mahesh et al., 2005; Venditto et al., 2006; Albunia et al., 2013), sensor (Mensitieri et al., 2003; Giordano et al., 2005; Cusano et al., 2006; Buono et al., 2009; Pilla et al., 2009; Erdogan et al., 2012) and catalysis (Buonerba et al., 2012) applications.

A first aim of the present paper is a deeper investigation of the scCO_2_ induced organoclay exfoliation, by a more complete X-ray diffraction characterization of organoclays before and after scCO_2_ treatments. The second aim of the paper is the preparation of composite aerogels containing large amounts of exfoliated organoclay as well as a nanoporous-crystalline polymer phase. The basic idea is that aerogel preparation processes, also based on scCO_2_ extraction, could help to maintain the clay exfoliation, which is generally lost in the nanocomposite processing (Manitiu et al., 2008). Advanced properties are pursued, through the synergy of aerogels and exfoliated clays. A montmorillonite (MMT) intercalated with dimethyl di(hydrogenated tallow) (2HT) ammonium cation was selected as the organoclay (OMMT in the following).

## Materials and methods

### Materials

Organically modified MMT, trade name Dellite® 67G, with 40 wt % of di(hydrogenated tallow)-dimethylammonium (2HT) was purchased from Laviosa Chimica Mineraria S.p.A.

The sPS used in this study was manufactured by Dow Chemicals under the trademark Questra 101. ^13^C nuclear magnetic resonance characterization showed that the content of syndiotactic triads (Grassi et al., 1987) was over 98%. The mass average molar mass obtained by gel permeation chromotography (GPC) in trichlorobenzene at 135°C was found to be *M*_*w*_ = 3.2 × 10^5^ g mol^−1^ with a polydispersity index *M*_*w*_/*M*_*n*_ = 3.9.

1,2-dichlorobenzene (DCB), used for clay dispersions, was purchased from Aldrich and used without further purification.

### Clay delamination and exfoliation by scCo_2_

Exfoliated clay samples were obtained by using a SFX 200 supercritical carbon dioxide extractor (ISCO Inc.). Organically modified clays (typically 10 mg in a 20 mL stainless steel vessel) were processed in scCO_2_ at 40°C and 200 bar, for 16 and 32 h under quiescent conditions, in order to promote the diffusion of the supercritical fluid into the clay interlayer space. The system was then rapidly depressurized (1 min) to atmospheric pressure and the expansion of the scCO_2_ between the layers caused the clay exfoliation.

### Preparation of sPS/clay gels

Dispersions of the clays in DCB were obtained with both as received and exfoliated samples. A clay dispersion was initially prepared by adding the appropriate clay amount in 5 ml of DCB. The mixtures were homogenized for 1 h under magnetic stirring and sonicated in a 5000 mL batch bath ultrasound (Badelin Sonorex RK 1028 H) for 1 h.

sPS/clay gels were prepared, in hermetically sealed test tubes, by heating the clay dispersions above the boiling point of the solvent until complete dissolution of the polymer and the appearance of a transparent and homogeneous solution had occurred. The hot solution was then cooled to room temperature, where gelation occurred. For instance, 655 mg of sPS and 5 mL of 2 wt % clay dispersion were mixed to obtain clay/polymer gels. The overall amount of polymer and clay in the gels was generally fixed to 10 wt %.

### Preparation of sPS/clay aerogels

Aerogels were obtained by treating sPS/clay gels with a SFX 200 supercritical carbon dioxide extractor (ISCO Inc.) using the following conditions: *T* = 40°C, *P* = 200 bar, extraction time *t* = 6 h. The prepared s-PS/clay aerogels present a weight composition ranging between 96/4 and 50/50. The aerogels, as prepared from gels with an overall polymer-clay content of 10 wt %, present a porosity close to 90%.

For monolithic aerogels with a regular cylindrical shape, the total porosity can be estimated from the mass/volume ratio of the aerogel. Then, the percentage of porosity *P* of the aerogel samples can be expressed as:

(1) $$P=100^{*}\text{​}\left[1-\left(\rho_{\text{app}}/\rho_{\text{pol}}\right)\right]$$

where ρ_pol_ is the density of the polymer matrix and ρ_app_ is the aerogel apparent density calculated from the mass/volume ratio of the monolithic aerogels.

### Characterization techniques

#### Wide angle x-ray diffraction

Wide-angle X-ray diffraction (WAXD) patterns with nickel filtered Cu-Kα radiation were obtained, with an automatic Bruker D8 Advance diffractometer, in reflection. The intensities of the WAXD patterns were not corrected for polarization and Lorentz factors, to allow an easier comparison with most literature data. The *D*_hkl_ correlation length of crystals was determined applying the Scherrer equation:

(2) $$D_{\text{hkl}}=K\lambda /\text{​}\left(\beta_{\text{hkl}}\text{cos }\theta_{\text{hkl}}\right)$$

where: *K* is the Scherrer constant, λ is the wavelength of the irradiating beam (1.5419 Å, CuKα), β_hkl_ is the width at half height, and θ_hkl_ is the diffraction angle. The instrumental broadening, *b*, was determined by obtaining a WAXD pattern of a standard silicon powder 325 mesh (purity >99%), under the same experimental conditions. For each observed reflection with β_hkl_ < 1°, the width at half height was evaluated by subtracting the unavoidable instrumental broadening of the closest silicon reflection from the experimental width at half height, β_hkl_, using the following relationship:

(3) $$\beta_{\text{hkl}}^{2}=\left(B_{\text{hkl}}^{2}-b^{2}\right)$$

#### Scanning electron microscopy

The internal morphology of the aerogels was characterized by means of a scanning electron microscope (SEM, Zeiss Evo50 equipped with an Oxford energy dispersive X-ray detector). Samples were prepared by fracturing small pieces of the monoliths in order to gain access to the internal part of the specimen. In fact, the external lateral surfaces of most samples were found to be flat and free of porosity. Low energy was used (5 keV) to obtain the highest possible surface resolution. Before imaging, all specimens were coated with gold using a VCR high resolution indirect ion-beam sputtering system. The samples were coated depositing approximately 20 nm of gold. The coating procedure was necessary to prevent surface charging during measurement and to increase the image resolution.

#### BET measurements

Nitrogen adsorption at liquid nitrogen temperature (77 K) was used to measure surface areas of OMMT and polymer/OMMT aerogels with a Nova Quantachrome 4200e instrument. Before the adsorption measurement, OMMT powders were degassed at 100°C under vacuum for 24 h, while polymer/clay aerogels were degassed at 40°C, in the same conditions. The surface area values were determined by using 11-point BET analysis.

#### Differential scanning calorimetry

The differential scanning calorimetry (DSC) of OMMT powders and polymer/OMMT aerogels was carried out under nitrogen from 0 to 300°C at a heating rate of 10°C/min on a TA instruments DSC 2920.

#### Dynamic-mechanical analysis

Dynamic-mechanical properties were studied using a Triton dynamic-mechanical thermal analyzer. The spectra were recorded in the three-point bending mode, on samples with the following dimensions: length 15 mm, width 10 mm, and thickness 2 mm. The modulus *E*′ was obtained, as a function of temperature, at a frequency of 1 Hz and an amplitude of 0.03 mm. The heating rate was 2°C/min in the range of 0, +100°C.

## Results and discussion

### OMMT exfoliation by scCo_2_

**Figure 1:**
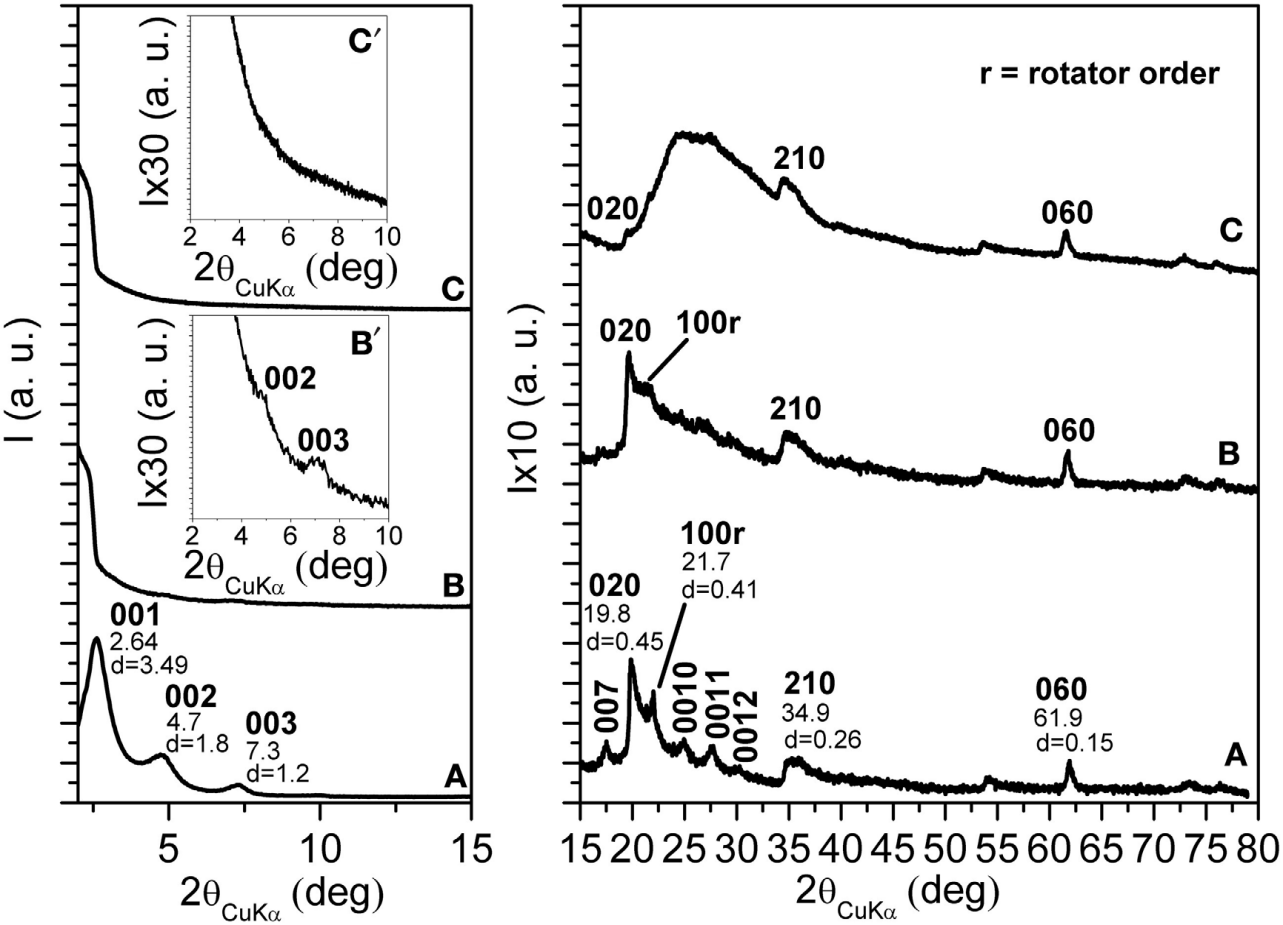
**X-ray diffraction (CuKα) patterns in the 2\upvartheta range 2–80° of MMT, as intercalated with 40 wt % of 2HT ammonium before (A) and after 16 h (B) and 32 h (C) scCO_2_ treatments**. The inset in **(B)** and **(C)** enlarges the 2\upvartheta range 2–10°.The Miller index 100r indicate the reflection relative to the rotator order of the long hydrocarbon chains within the interlayer space.

**Figure 2:**
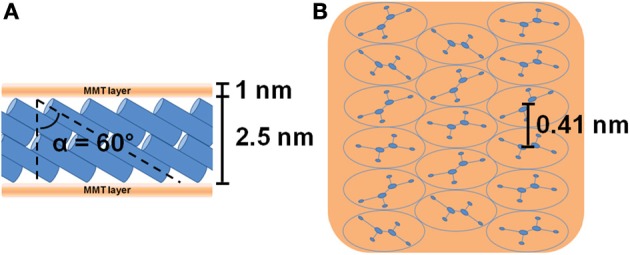
**Schematic projections parallel (A) and perpendicular (B) to the clay layers of the starting MMT/2HT (OMMT) intercalate structure**. The hydrocarbon tails of the ammonium cations are represented as cylinders in the lateral view **(A)** and as ellipses in the top view **(B)**. The distance between the axes of adjacent cylinders (0.41 nm) is shown in **(B)** while the definition of the alkyl chains tilt angle is shown in **(A)**.

The X-ray diffraction (CuKα) pattern in the 2\upvartheta range 2–80° of the OMMT, with 40 wt % of 2HT, is reported in Figure 1A. Besides many 00*l* reflections (up to *l* = 12) that indicate a high degree of order perpendicular to the clay layers and an interlayer spacing of 3.5 nm, the pattern shows well defined weak peaks, corresponding to the typical 020, 210, and 060 in-plane MMT periodicities (Galimberti et al., 2007; Powder diffraction database 70-2151 International Centre for Diffraction Data, 1999). It is worth adding that a well defined narrow peak is also present at 2\upvartheta = 21.7°, corresponding to *d* = 0.41 nm, i.e., the distance between long hydrocarbon chains in their rotator order, analogous to those observed for long-chain alkylammoniums intercalated in anionic clays (layered double hydrotalcite, LDH) (Itoh et al., 2003) as well as in graphite oxide (Mauro et al., 2013) The thickness of the clay layer (≈1 nm) and the length of the alkylammoniums (≈5 nm) (Osman et al., 2002) indicate that the tilting angle of the hydrocarbon chains is not far from α = 60°. Hence, the X-ray diffraction pattern of Figure 1A indicates the presence of a MMT/2HT intercalate structure, whose schematic projections, parallel and perpendicular to the clay layers, are shown in Figures 2A,B, respectively.

The X-ray diffraction patterns of the OMMT of Figure 1A, after short term and long term treatments by scCO_2_ are shown in Figures 1B,C, respectively. For the intermediate pattern of Figure 1B, the intensities of the 00*l* peaks as well as of the rotator order peak (100r) are strongly reduced (see also the inset of Figure 1B). The in-plane 020, 210, and 060 peaks maintain their intensity and narrowness. This indicates that short term scCO_2_ treatments lead to a nearly complete clay exfoliation with maintenance of the in-plane order.

In agreement with previous results (Manitiu et al., 2008), the X-ray diffraction pattern of the OMMT, after long-term scCO_2_ treatments, does not show anymore the 00*l* reflections (see also the inset of Figure 1C): clay exfoliation is achieved. It is worth adding that the in-plane 020, 210, and 060 reflections are still present, although become less intense than a broad amorphous halo that appears in the 2θ range 20–30°. This amorphous halo can be attributed to a loss of order in the stacking of the clay layers, also associated with a complete loss of order in the packing of the hydrocarbon tails.

In summary, the described long-term scCO_2_ treatments lead to exfoliation of the OMMT, and to a complete loss of long-range lateral order of the hydrocarbon tails of the cationic surfactant. The maintenance of hk0 reflections (mainly of the isolated 060 reflection), not yet reported in the literature, assures the maintenance of a long-range order in the clay layers. In this respect, it is worth adding that the half-height width of the 060 reflection, after exfoliation, remains equal to 0.45° indicating a correlation length *D*_060_ = 28 nm.

**Figure 3:**
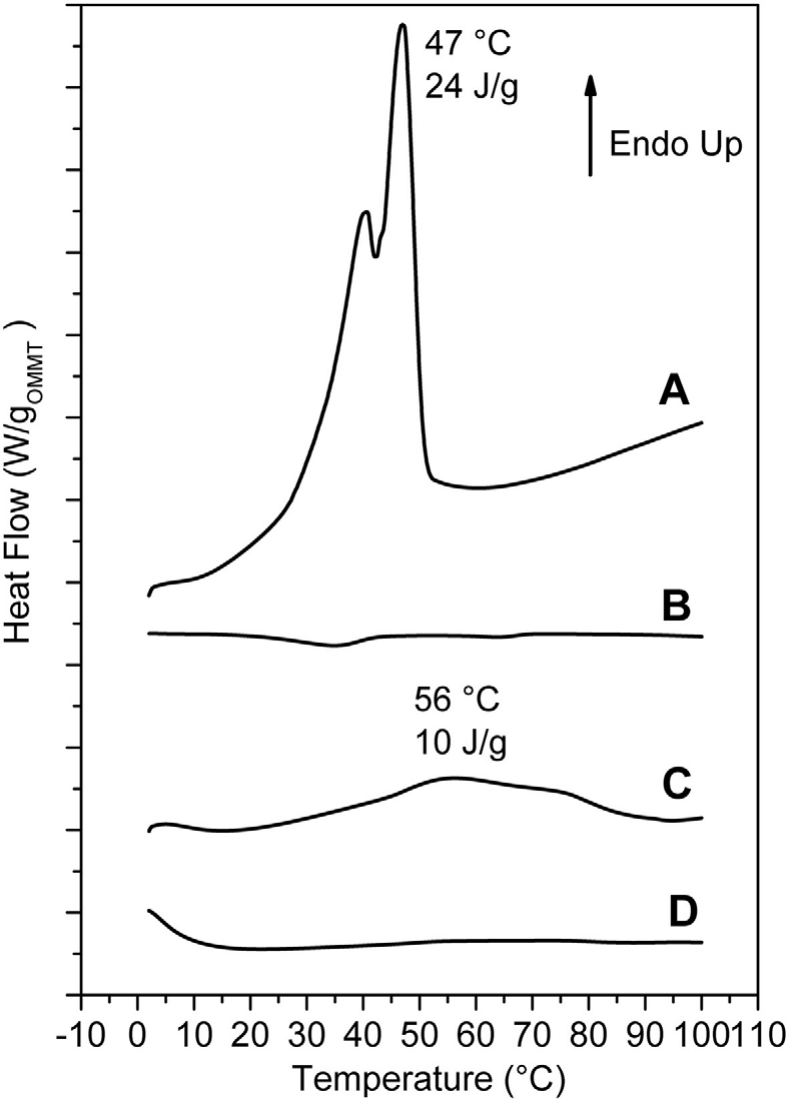
**DSC heating scans of: (A) as received MMT/2HT (intercalated); (B) MMT/2HT after treatment by scCO_2_ (exfoliated); (C,D) aerogels with 90% of porosity, with 50/50 weight ratio of s-PS/as received OMMT (C) and of sPS/exfoliated OMMT (D)**.

Relevant additional information, relative to the as received and scCO_2_-treated OMMT, can be obtained by DSC scans (Figure 3). The scan of the as received OMMT (Figure 3A) presents a reversible transition nearly located at 44°C (ΔH ≈26 J/g) that corresponds to the loss of rotator order of the hydrocarbon tails of the cations intercalated in the interlayer space. In this respect, it is worth adding that endothermic peaks corresponding to the loss of order in the packing of the hydrocarbon tails have been observed, in the temperature range 25–80°C, not only for cationic organoclays, (Cipolletti et al., 2013) but also for other organically modified layered inorganic structures (Ide and Ogawa, 2006) as well as for graphite oxide intercalation compounds (Mauro et al., 2013). The DSC scan of the scCO_2_ treated OMMT does not present any thermal transition in the considered temperature range (Figure 3B) and hence indicates the loss of 3D order in the packing of the hydrocarbon tails of the ammonium surfactant, which is compatible with clay exfoliation.

The overall information arising from X-ray diffraction and DSC characterization allows to conclude that the as received and scCO_2_ treated OMMT can be described as intercalated and exfoliated OMMT, respectively.

### Monolithic nanoporous-crystalline s-PS aerogels with large OMMT content

Monolithic composite aerogels, filled with large fractions of intercalated and exfoliated OMMT, have been prepared by using an s-PS matrix. This polymer choice is mainly due to the ability of s-PS to produce monolithic aerogels in a very broad range of porosity (from 50% up to 99%). An additional reason for this choice is the easy obtainment of aerogels exhibiting s-PS nanoporous-crystalline δ (Daniel et al., 2005, 2008) or ε (Daniel et al., 2009) phases.

Aerogels with a porosity of nearly 90% were obtained by scCO_2_ extraction of gels with a DCB content of 90 wt % and with different s-PS/OMMT weight ratios. For all aerogels with polymer/OMMT ratio equal or higher than 80/20, monolithic structures were obtained. Moreover, as usual for s-PS based aerogels, (Daniel et al., 2005, 2008, 2009, 2012, 2013a, b) the size and shape of s-PS/clay aerogels are essentially the same of the precursor gels. Aerogels with a 50/50 polymer/OMMT ratio are brittle and are generally obtained as powders.

**Figure 4:**
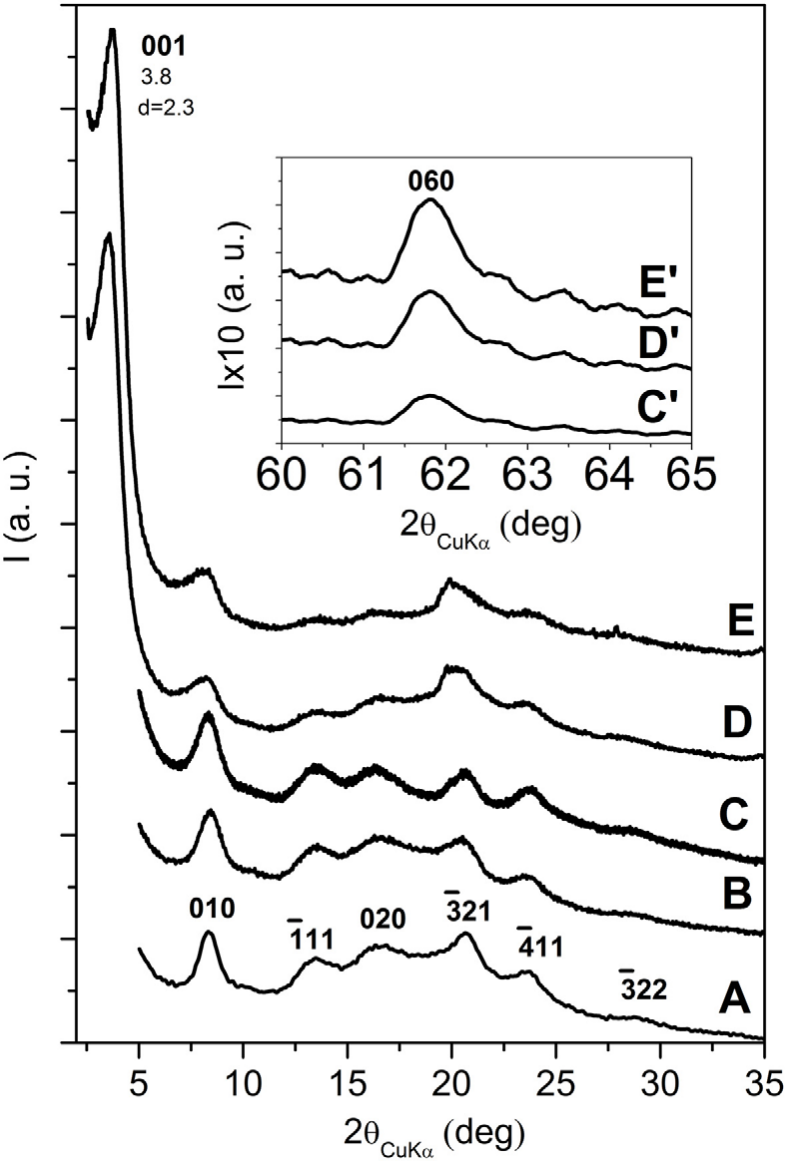
**X-ray diffraction (CuKα) patterns in the 2\upvartheta range 2–35° of s-PS aerogels with as received OMMT, as obtained from gels with a solvent content of 90 wt % and presenting different polymer/OMMT weight ratios: (A) 100/0; (B) 96/4; (C) 92/8; (D) 80/20; (E) 50/50**. The insets C′, D′ and E′ enlarge the 060 in-plane reflection of the clay. The Miller indexes of the main reflections of the nanoporous-crystalline δ form of s-PS are indicated in **(A)**.

**Figure 5:**
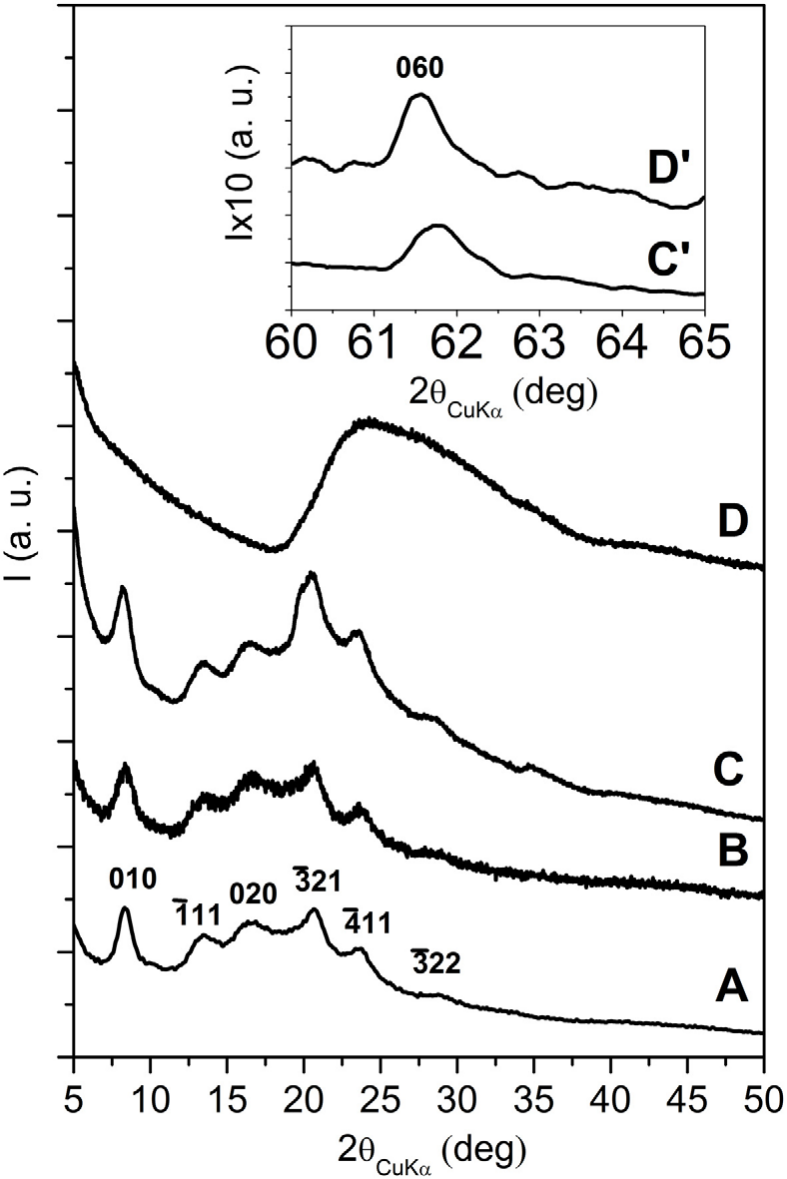
**X-ray diffraction (CuKα) patterns in the 2\upvartheta range 5–50° of s-PS aerogels with exfoliated OMMT, as obtained from gels with a solvent content of 90 wt % and presenting different polymer/OMMT weight ratios: (A) 100/0; (B) 95/5; (C) 80/20; (D) 50/50**. The inset C′, D′ enlarges the 060 in-plane reflection of the clay.

X-ray diffraction (CuKα) patterns of s-PS based aerogels, containing intercalated and exfoliated OMMT, are shown in Figures 4, 5, respectively.

All patterns of Figure 4 show the typical reflections of the nanoporous-crystalline δ form. In particular, the isolated intense 010 reflection is always clearly apparent and located at 2θ ≈8.4°. The 00*l* reflections of the OMMT are not detected for the aerogels with low clay content (4 and 8 wt %) while for higher clay contents (20 and 50 wt %) a narrow and intense 001 reflection is present, while the 002 and 003 reflections of the starting clay have disappeared. Moreover, the 001 reflection is markedly shifted with respect to its original position (from 2θ = 2.6° up to 2θ = 3.8°), indicating a decrease of the interlayer spacing from *d* = 3.5 nm down to *d* = 2.3 nm.

The results of Figures 4A–C suggest that the aerogel preparation procedure involving scCO_2_ extraction, for low OMMT content, could lead to clay exfoliation, as already observed for scCO_2_ treatment of the neat OMMT in Figure 1. Figures 4C,D show that, for high OMMT content in the aerogels, the used procedure is not suitable to generate OMMT exfoliation but, on the contrary, a reduction of the interlayer spacing is observed. An analogous phenomenon of reduction of interlayer spacing has been recently observed for organoclay extraction with different solvents, like e.g., ethyl acetate (Cipolletti et al., 2013). As suggested in that paper, the observed reduction of basal spacing can be attributed to the extraction of excess cationic surfactant, not being ionically bonded to the negatively charged clay layers, but being simply included in the interlayer space by non-bonded interactions and contributing to the crystalline order of the hydrocarbon tails.

Additional information on the structural organization in the s-PS/OMMT aerogels comes from DSC analyses. In particular, DSC heating scan of a 50/50 by wt. s-PS/as-received-clay aerogel is shown in Figure 3C. The endothermic peak, corresponding to loss of rotator order in the interlayer spacing (Figure 1A) becomes broader and its maximum is shifted up to 50–60°C, with only a minor reduction of the related enthalpy (Δ H_r_ ≈10 J/g ≈20 J/g_OMMT_).

The combined information of the X-ray diffraction patterns of Figures 4C,D and the DSC scans of Figure 3C indicates that, for high clay content, the aerogel preparation procedure brings to a reduction of the OMMT basal spacing (*d*_001_) from 3.5 nm down to 2.3 nm, with only partial loss of the hydrocarbon rotator order in the interlayer space.

The X-ray diffraction patterns of the s-PS aerogels prepared with the exfoliated OMMT (Figure 5), independently of the aerogel composition, do not show 00*l* clay reflections, while show the isolated weak 060 in-plane clay reflection (as shown by the inset of Figures 5C′,D′). This clearly indicates that the gel and aerogel preparation procedures, also for high clay concentrations, allow to maintain clay exfoliation without re-aggregation, as instead observed for other common polymer processing (Nguyen and Baird, 2007; Manitiu et al., 2008).

In this respect, it is worth citing that X-ray diffraction patterns of polymer-clay aerogels as obtained by freeze-drying of polymer solutions including clays (Bandi et al., 2005; Pojanavaraphan et al., 2010, 2011; Wang et al., 2013) show the presence of 00*l* clay reflections, (Pojanavaraphan et al., 2010; Wang et al., 2013), which exclude the occurrence of exfoliation.

The patterns of Figure 5 also show that s-PS is generally crystallized in the nanoporous δ form (Figures 5A–C) while, for the 50/50 polymer/exfoliated-OMMT aerogel, the s-PS crystallization does not occur (broad amorphous halo of Figure 5D). This is probably due to the good dispersion of a large amount of exfoliated OMMT, leading to a diluting effect on sPS that reduces its crystallization kinetics. This loss of polymer crystallinity leads to a loss of the typical fibrillar structure, which in turn allows rationalizing the loss of monolithic structure.

### Comparison between aerogels with intercalated and exfoliated OMMT

This section presents a strict comparison between properties of s-PS monolithic aerogels exhibiting a porosity of 90% and a OMMT content of 20 wt %, as obtained by using intercalated or exfoliated OMMT, that present the X-ray diffraction patterns shown in Figures 4D, 5C, respectively.

**Figure 6:**
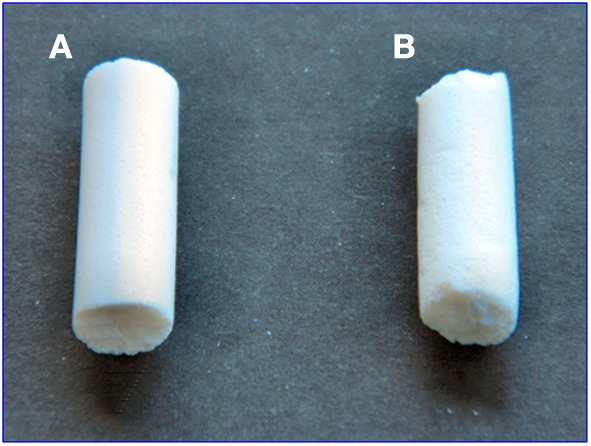
**Photographs of cylindrical monolithic (diameter of 7 mm) s-PS/OMMT aerogels, with porosity *P* = 90%, as obtained by scCO_2_ drying and exhibiting a 80/20 weight ratio: (A) with exfoliated clay; (B) with intercalated clay**. The shown aerogels essentially present the same size and shape of the precursor gels.

On the basis of quantitative evaluations on the X-ray diffraction patterns, the two aerogels present similar degree of polymer crystallinity (≈40%). However, aerogels with the exfoliated clay (Figure 6A) are much more homogeneous than aerogels obtained with the intercalated OMMT (Figure 6B), which clearly present rough surfaces.

**Figure 7:**
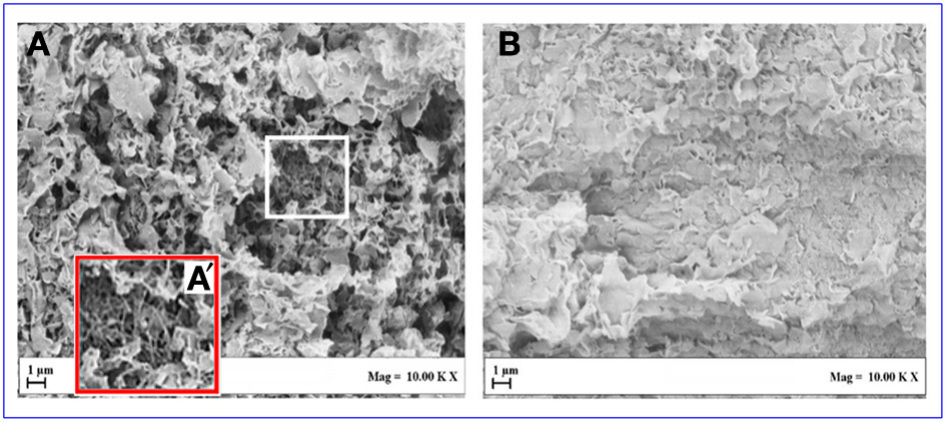
**SEM of aerogels with porosity *P* = 90%, having 80/20 polymer/OMMT weight ratio: (A,A′) with exfoliated OMMT; (B) with intercalated OMMT**.

Also the SEM images of the two aerogels are completely different. In fact, the SEM of the aerogel including the intercalated OMMT is dominated by the micrometric OMMT particles (Figure 7B) while the SEM of the aerogel including the exfoliated OMMT (Figure 7A) clearly shows both nanometric clay platelets and nanometric s-PS fibrils (Figure 7A′).

The results of the SEM analyses suggest that also the large difference in the visual appearance between the two aerogels of Figure 6 could be due to micrometric and nanometric size of intercalated and exfoliated clays, respectively.

DMA analyses indicate that aerogels based on the exfoliated clay present an elastic modulus definitely higher than for those based on intercalated OMMT (36 MPa vs. 15 MPa).

Surface areas *S*_BET_, as obtained by N_2_ adsorption data at 77 K, for the intercalated and exfoliated OMMT, as well as those of the corresponding aerogels, are compared in Table 1. For the sake of comparison, *S*_BET_ of the neat s-PS aerogel presenting the same porosity is shown in the last row of Table 1. As well known, s-PS aerogels exhibit high surface areas, mainly associated with the crystalline cavities of the δ crystalline phase, but also associated with the amorphous aerogel porosity (Daniel et al., 2005, 2008, 2009, 2012, 2013a, b). In agreement with literature data, (Park et al., 2011) *S*_BET_ of the OMMT is rather low and is substantially increased for the exfoliated OMMT (*S*_BET_ = 18 m^2^/g). The s-PS/exfoliated-clay aerogels present values of *S*_BET_ (281 m^2^g^−1^) much higher than those of the s-PS/intercalated-clay aerogels (166 m^2^g^−1^) and not far from those observed for pure s-PS aerogels (312 m^2^g^−1^). This indicates that, also for this high OMMT content (20 wt %), the exfoliated clay not only does not disturb the formation of the nanoporous crystalline phase but also does not alter the amorphous aerogel porosity.

**Table 1:** **Total surface area (*S*_BET_) of OMMT samples and of aerogels with porosity *P* = 90%, having 80/20 polymer/OMMT weight ratio**.

**Sample**	***S*_BET_^a^ (m^2^g^−1^)**
Intercalated OMMT	10
Exfoliated OMMT (scCO_2_ treated)	18
sPS/intercalated-OMMT, 80/20 aerogel	166
sPS/exfoliated-OMMT, 80/20 aerogel	281
Aerogel δ sPS	312

a Total area evaluated following the BET model in the standard 0.05 < P/P_0_ < 0.3 pressure range.

**Figure 8:**
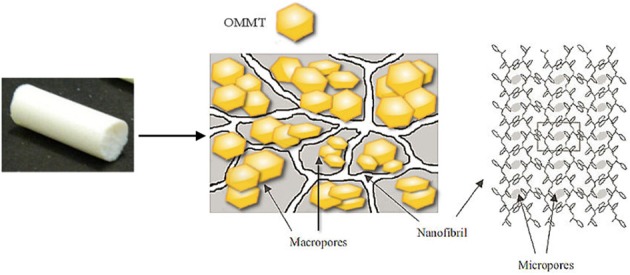
**Schematic representation of the sPS/exfoliated-OMMT aerogels**.

A schematic representation of the sPS/exfoliated-OMMT aerogels is shown in Figure 8.

## Conclusions

A thorough investigation of scCO_2_-induced exfoliation of OMMTs has been conducted mainly by X-ray diffraction and DSC characterization techniques. The starting material is a MMT intercalated with ammonium cations bearing two long hydrocarbon tails. Suitable scCO_2_ treatments led to exfoliation of the OMMT and also led to a complete loss of long-range order in the packing of the hydrocarbon tails of the cationic surfactant. The maintenance of hk0 reflections (mainly of the isolated 060 reflection), not yet reported in the literature, assures the maintenance of a long-range order in the clay layers. DSC scans of the intercalated OMMT present a reversible transition that corresponds to the loss of rotator order of the hydrocarbon chains in the interlayer spacing while those of the exfoliated OMMT do not present any thermal transition up to 100°C. This confirms the absence of any 3-D order for the exfoliated clay.

Monolithic composite aerogels, filled with large amounts of both intercalated and exfoliated OMMT, have been prepared, starting from s-PS-based gels. In particular, for aerogels with high content of the intercalated OMMT, the preparation procedure brings to a reduction of the basal spacing (*d*_001_) from 3.5 nm down to 2.3 nm, with only partial loss of the hydrocarbon rotator order in the interlayer space. For aerogels with high content of the exfoliated OMMT, the gel and aerogel preparation procedures allow to maintain clay exfoliation without re-aggregation, as instead observed for other common polymer processing.

A strict comparison between s-PS monolithic aerogels with a porosity of 90% and a OMMT content of 20 wt %, as obtained by using intercalated or exfoliated OMMT, has been also reported. Although the two aerogels present similar degree of polymer crystallinity (≈40%) as well as the same polymer crystalline form (the nanoporous-crystalline δ form), aerogels with the exfoliated OMMT are much more homogeneous than aerogels with the intercalated OMMT. This difference, clearly apparent both on visual inspection as well as on SEM analysis, is due to micrometric and nanometric size of intercalated and exfoliated clays, respectively. Aerogels based on the exfoliated clay also present elastic modulus definitely higher than those based on intercalated OMMT. Moreover, s-PS/exfoliated-clay aerogels present values of surface area (281 m^2^g^−1^) much higher than those of the s-PS/intercalated-clay aerogels (166 m^2^g^−1^) and not far from those observed for pure s-PS aerogels (312 m^2^g^−1^). This indicates that, also for high content, the exfoliated clay does not alter the aerogel porosity.

The improvement of properties such as the modulus and the surface area is definitely of interest in view of potential applications of aerogels with exfoliated OMMT, for example for achieving relevant transport properties with extremely light materials. Moreover, the clay rich aerogels (e.g., 50/50, w/w), could be helpful to an easier handling of exfoliated OMMT, removing the risks connected with inhalable nanoparticles.

### Conflict of interest statement

The authors declare that the research was conducted in the absence of any commercial or financial relationships that could be construed as a potential conflict of interest.

## References

[B1] Albunia A. R. Rizzo P. Guerra G. (2013). Control of guest transport in polymer films by structure and orientation of nanoporous-crystalline phases. Polymer 54, 1671–1678 10.1016/j.polymer.2013.01.027

[B2] Alexandre M. Dubois P. (2000). Polymer layered silicate nanocomposites: preparation, properties and uses of a new class of materials. Mater. Sci. Eng. R. Rep. 28, 1–63 10.1016/S0927-796X(00)00012-7

[B3] Anderson M. L. Stroud R. M. Rolison D. R. (2002). Enhancing the activity of fuel-cell reactions by designing three-dimensional nanostructered architectures, catalyst-modified carbon-silica composite aerogels. Nano Lett. 2, 235–240 10.1021/nl015707d

[B4] Baker K. C. Manitiu M. Bellair R. Gratopp C. A. Herkowitz H. N. Kannan R. M. (2011). Supercritical carbon dioxide processed resorbable polymer nanocomposite bone graft substitutes. Acta Biomater. 7, 3382–3389 10.1016/j.actbio.2011.05.014 21640204

[B5] Bandi S. Bell M. Schiraldi D. A. (2005). Temperature-responsive clay aerogel-polymer composites. Macromolecules 38, 9216–9220 10.1021/ma051698+

[B6] Bergaya F. Lagaly G. (2013). Handbook of Clay Science. 2nd Edn. Elsevier, Amsterdam

[B7] Bergaya F. A. (2008). Layered clay minerals: basic research and innovative composite applications. Microp. Mesop. Mater. 107, 141–148 10.1016/j.micromeso.2007.05.064

[B8] Buonerba A. Cuomo C. Ortega Sánchez S. Canton P. Grassi A. (2012). Gold nanoparticles incarcerated in nanoporous syndiotactic polystyrene matrices as new and efficient catalysts for alcohol oxidations. Chem. Eur. J. 18, 709–715 10.1002/chem.201101034 22162281

[B9] Buono A. Rizzo P. Immediata I. Guerra G. (2009). Detection and memory of nonracemic molecules by a racemic host polymer film. J. Am. Chem. Soc. 129, 10992–10993 10.1021/ja0732936 17705489

[B10] Chen B. Evans J. R. G. Greenwell H. C. Boulet P. Coveney P. V. Bowden A. A. (2008). A critical appraisal of polymer-clay nanocomposites. Chem. Soc. Rev. 37, 568–594 10.1039/b702653f 18224264

[B11] Chen C. Samaniuk J. Baird D. G. Devoux G. Zhang M. Moore R. B. (2012). The preparation of nano-clay/polypropylene composite materials with improved properties using supercritical carbon dioxide and a sequential mixing technique. Polymer 53, 1373–1382 10.1016/j.polymer.2012.01.049

[B12] Choi Y. S. Ham H. T. Chung I. (2004). Effect of monomers on the basal spacing of sodium montmorillonite and the structures of polymer-clay nanocomposites. J. Chem. Mater. 16, 2522–2529 10.1021/cm0348601

[B13] Chou C. C. Lin J. (2005). One-step exfoliation of montmorillonite via phase inversion of amphiphilic copolymer emulsion. Macromolecules 38, 230–233 10.1021/ma047761x

[B14] Cipolletti V. Galimberti M. Guerra G. Mauro M. (2013). Organoclays with hexagonal rotator order for the paraffinic chains of the compensating cation. Implications on the structure of clay polymer nanocomposites. Appl. Clay Sci. 10.1016/j.clay.2013.11.001

[B15] Cusano A. Iadicicco A. Pilla P. Contessa L. Campopiano S. Cutolo A. (2006). Coated long-period fiber gratings as high-sensitivity optochemical sensors. J. Lightw. Technol. 24, 1776–1786 10.1109/JLT.2006.871128

[B16] Daniel C. Alfano D. Venditto V. Cardea S. Reverchon E. Larobina D. (2005). Aerogels with microporous crystalline host phase. Adv. Mater. 17, 1515–1518 10.1002/adma.200401762

[B17] Daniel C. Giudice S. Guerra G. (2009). Syndiotatic polystyrene aerogels with beta, gamma and epsilon crystalline phases. Chem. Mater. 21, 1028–1034 10.1021/cm802537g

[B18] Daniel C. Longo S. Cardea S. Vitillo J. G. Guerra G. (2012). Monolithic nanoporous–crystalline aerogels based on PPO. RSC Adv. 2, 12011–12018 10.1039/c2ra22325b

[B19] Daniel C. Longo S. Ricciardi R. Reverchon E. Guerra G. (2013a). Monolithic nanoporous crystalline aerogels. Macromol. Rapid. Commun. 34, 1194–1207 10.1002/marc.201300260 23913316

[B20] Daniel C. Zhovner D. Guerra G. (2013b). Thermal stability of nanoporous crystalline and amorphous phases of poly(2, 6-dimethyl-1, 4-phenylene)oxide. Macromolecules 46, 449–454 10.1021/ma302227q

[B21] Daniel C. Longo S. Vitillo J. G. Fasano G. Guerra G. (2011). Nanoporous crystalline phases of poly(2, 6-dimethyl-1, 4-phenylene)oxide. Chem. Mater. 23, 3195–3200 10.1021/cm200546r

[B22] Daniel C. Sannino D. Guerra G. (2008). Syndiotactic polystyrene aerogels: adsorption in amorphous pores and absorption in crystalline nanocavities. Chem. Mater. 20, 577–582 10.1021/cm702475a

[B23] De Rosa C. Guerra G. Petraccone V. Pirozzi B. (1997). Crystal structure of the emptied clathrate form (δ form) of syndiotactic polystyrene. Macromolecules 30, 4147–4152 10.1021/ma970061q

[B24] Erdogan M. Ozbek Z. Capan R. Yagci Y. (2012). Characterization of polymeric LB thin films for sensor applications. J. Appl. Polym. Sci. 123, 2414–2422 10.1002/app.34793

[B25] Feng-hua S. Han-xiong H. Yang Z. (2011). Microstructure and mechanical properties of polypropylene/poly(ethylene-co-octene copolymer)/clay ternary nanocomposites prepared by melt blending using supercritical carbon dioxide as a processing aid. Compos. Part B 42, 421–428 10.1016/j.compositesb.2010.12.005

[B26] Galimberti M. (ed.) (2011). Rubber Clay Nanocomposites-Science, Technology and Applications. New York, NY: Wiley and Sons 10.1002/9781118092866

[B27] Galimberti M. Giudice S. Cipolletti V. Guerra G. (2010). Control of organoclay structure in hydrocarbon polymers. Polym. Adv. Tech. 21, 1–6 10.1002/pat.1757

[B28] Galimberti M. Lostritto A. Spatola A. Guerra G. (2007). Clay delamination in hydrocarbon rubbers. Chem. Mater. 19, 2495–2499 10.1021/cm062782m18572583

[B29] Galimberti M. Senatore S. Conzatti L. Costa G. Giuliano G. Guerra G. (2009). Formation of clay intercalates with organic bilayers in hydrocarbon polymers. Polym. Adv. Technol. 20, 135–142 10.1002/pat.128723540870

[B30] Galizia M. Daniel C. Fasano G. Guerra G. Mensitieri G. (2012). Gas sorption and diffusion in amorphous and semicrystalline nanoporous poly(2, 6-dimethyl-1, 4-phenylene)oxide. Macromolecules 45, 3604–3615 10.1021/ma3000626

[B31] Giordano M. Russo M. Cusano A. Mensitieri G. Guerra G. (2005). Syndiotactic polystyrene thin film as sensitive layer for an optoelectronic chemical sensing device. Sens. Actuators B 109, 177–184 10.1016/j.snb.2004.02.053

[B32] Gowd E. B. Shibayama N. Tashiro K. (2006). Structural changes in thermally induced phase transitions of uniaxially oriented δ_e_ form of syndiotactic polystyrene investigated by temperature-dependent measurements of X-Ray fiber diagrams and polarized infrared spectra. Macromolecules 39, 8412–8418 10.1021/ma061659d

[B33] Grassi A. Pellecchia C. Longo P. Zambelli A. (1987). Selective synthesis of syndiotactic polystyrene. Gazz. Chim. Ital. 117, 249–250 22162281

[B34] Horsch S. Serhatkulu G. Gulari E. Kannan R. M. (2006). Supercritical CO_2_ dispersion of nano-clays and clay/polymer nanocomposites. Polymer 47, 7485–7496 10.1016/j.polymer.2006.08.048

[B35] Ide Y. Ogawa M. (2006). Preparation and some properties of organically modified layered alkali titanates with alkylmethoxysilanes. J. Colloid. Interface Sci. 296, 141–149 10.1016/j.jcis.2005.08.058 16169002

[B36] Itoh T. Ohta N. Shichi T. Yui T. Takagi K. (2003). The self-assembling properties of stearate ions in hydrotalcite clay composites. Langmuir 19, 9120–9126 10.1021/la0302448

[B37] Kistler S. S. (1931). Coherent expanded aerogels and jellies. Nature 127, 741 10.1038/127741a0

[B38] Kojima Y. Usuki A. Kawasumi M. Okada A. Fukushima Y. Kurauchi (1993). Mechanical properties of nylon 6-clay hybrid. J. Mater. Res. 8, 1179–1185 10.1557/JMR.1993.1185

[B39] Kucheyev S. O. Stadermann M. Shin S. J. Satcher J. H. Gammon S. A. Letts S. A. (2012). Super-compressibility of ultralow–density nanoporous silica. Adv. Mater. 24, 776–780 10.1002/adma.201103561 22228389

[B40] LeBaron P. C. Wang Z. Pinnavaia T. J. (1999). Polymer-layered silicate nanocomposites: an overview. Appl. Clay Sci. 15, 11–29 10.1016/S0169-1317(99)00017-4

[B41] Longo S. Vitillo J. G. Daniel C. Guerra G. (2013). Monolithic aerogels Based on poly(2, 6-diphenyl-1, 4-phenylene oxide) and syndiotactic polystyrene. ACS Appl. Mater. Interfaces 5, 5493–5499 10.1021/am400592z 23701278

[B42] Ma J. Bilotti E. Peijs T. Darr J. A. (2007). Preparation of polypropylene/sepiolite nanocomposites using supercritical CO_2_ assisted mixing. Eur. Polym. J. 43, 4931–4939 10.1016/j.eurpolymj.2007.09.010

[B43] Mahesh K. P. O. Sivakumar M. Yamamoto Y. Tsujita Y. Yoshimizu H. Okamoto S. (2005). Structure and properties of the mesophase of syndiotactit polystyrene. J. Membrane Sci. 262, 11–19 10.1016/j.memsci.2005.04.002

[B44] Malik S. Roizard D. Guenet J. M. (2006). Multiporous material from fibrillar syndiotactic polystyrene intercalates. Macromolecules 39, 5957–5959 10.1021/ma060770g

[B45] Manfredi C. Del Nobile M. A. Mensitieri G. Guerra G. Rapacciuolo M. (1997). Vapor sorption in emptied clathrate samples of syndiotactic polystyrene. J. Polym. Sci. Polym. Phys. Ed. 35, 133–140 10.1002/(SICI)1099-0488(19970115)35:1<133::AID-POLB11>3.0.CO;2-E

[B46] Manias E. Touny A. Wu L. Strawhecker K. Lu B. Chung T. C. (2001). Polypropylene/Montmorillonite nanocomposites. Review of the synthetic routes and materials properties. Chem. Mater. 13, 3516–3523 10.1021/cm0110627

[B47] Manitiu M. Bellair R. J. Horsch S. Gulari E. Kannan R. M. (2008). Supercritical carbon dioxide-processed dispersed polystyrene-clay nanocomposites. Macromolecules 41, 8038–8046 10.1021/ma801339g21640204

[B48] Mauro M. Maggio M. Cipolletti V. Galimberti M. Longo P. Guerra G. (2013). Graphite oxide intercalation compounds with rotator hexagonal order in the intercalated layers. Carbon 61, 395–403 10.1016/j.carbon.2013.05.023

[B49] Mensitieri G. Venditto V. Guerra G. (2003). Polymeric sensing films absorbing organic guests into a nanoporous host crystalline phase. Sens. Actuators B 92, 255–261 10.1016/S0925-4005(03)00273-9

[B50] Milano G. Venditto V. Guerra G. Cavallo L. Ciambelli P. Sannino D. (2001). Shape and volume of cavities in thermoplastic molecular sieves based on syndiotactic polystyrene. Chem. Mater. 13, 1506–1511 10.1021/cm001089a

[B51] Musto P. Mensitieri G. Cotugno S. Guerra G. Venditto V. (2002). Probing by time-resolved FTIR spectroscopy mass transport, molecular interactions, and conformational ordering in the system chloroform-syndiotactic polystyrene. Macromolecules 35, 2296–2304 10.1021/ma011684d

[B52] Nguyen Q. T. Baird D. G. (2007). An improved technique for exfoliating and dispersing nanoclay particles into polymer matrices using supercritical carbon dioxide. Polymer 48, 6923–6933 10.1016/j.polymer.2007.09.015

[B53] Osman M. A. Ernst M. Meier B. H. Suter U. W. (2002). Structure and molecular dynamics of alkane monolayers self-assembled on mica platelets. J. Phys. Chem. B 106, 653–662 10.1021/jp0132376

[B54] Park Y. Ayoko G. A. Frost R. L. (2011). Characterisation of organiclays and adsorption of p-nitrophenol: environmental application. J. Colloid Interface Sci. 360, 440–456 10.1016/j.jcis.2011.04.085 21600587

[B55] Paul D. R. Robeson L. M. (2008). Polymer nanotechnology: nanocomposites. Polymer 49, 3187–3204 10.1016/j.polymer.2008.04.017

[B56] Petraccone V. Ruiz de Ballesteros O. Tarallo O. Rizzo P. Guerra G. (2008). Nanoporous polymer crystals with cavities and channels. Chem. Mater. 20, 3663–3668 10.1021/cm800462h

[B57] Pilla P. Cusano A. Cutolo A. Giordano M. Mensitieri G. Rizzo P. (2009). Molecular sensing by nanoporous crystalline polymers. Sensors 9, 9816–9857 10.3390/s91209816 22303150 PMC3267198

[B58] Pojanavaraphan T. Liu L. Ceylan D. Okay O. Magaraphan R. Schiraldi. D. A. (2011). Solution cross-linked natural rubber (NR)/clay aerogel composites. Macromolecules 44, 923–931 10.1021/ma102443k

[B59] Pojanavaraphan T. Schiraldi D. A. Magaraphan R. (2010). Mechanical, rheological, and swelling behavior of natural rubber/montmorillonite aerogels prepared by freeze-drying. Appl. Clay Sci. 50, 271–279 10.1016/j.clay.2010.08.020

[B60] Powder diffraction database 70-2151 International Centre for Diffraction Data. (1999). PCPDF win. Version 2.02.

[B61] Ray S. S. Okamoto M. (2003). Polymer/layered silicate nanocomposites: a review from preparation to processing. Prog. Polym. Sci. 28, 1539–1641 10.1016/j.progpolymsci.2003.08.002

[B62] Rizzo P. Daniel C. De Girolamo Del Mauro A. Guerra G. (2007). New host polymeric framework and related polar guest cocrystals. Chem. Mater. 19, 3864–3866 10.1021/cm071099c

[B63] Robello D. R. Yamaguchi N. Blanton T. Barnes C. (2004). Spontaneous formation of an exfoliated polystyrene-clay nanocomposite using a star-shaped polymer. J. Am. Chem. Soc. 126, 8118–8119 10.1021/ja048211h 15225044

[B64] Samaniuk J. Litchfield D. Baird D. (2009). Improving the exfoliation of layered silicate in a poly(ethylene terephthalate) matrix using supercritical carbon dioxide. Polym. Eng. Sci. 49, 2329–2341 10.1002/pen.21482

[B65] Schaefer D. W. Keefer K. D. (1986). Restructuring of colloidal silica aggregates. Phys. Rev. Lett. 56, 2199 10.1103/PhysRevLett.56.2199 10033272

[B66] Tarallo O. Petraccone V. Daniel C. Fasano G. Rizzo P. Guerra G. (2012). A chiral co-crystalline form of poly(2, 6-dimethyl-1, 4-phenylene)oxide (PPO). J. Mater. Chem. 22, 11672–11680 10.1039/c2jm30907f

[B67] Thompson M. R. Balogh M. P. Speer R. L. Fasulo P. D. Rodgers W. R. (2009). In *situ* X-ray diffraction studies of alkyl quaternary ammonium montmorillonite in a CO_2_ enviroment. J. Chem. Phys. 130, 044705 10.1063/1.3065980 19191402

[B68] Treece M. A. Oberhauser J. P. (2007). Processing of polypropylene-clay nanocomposites: single screw extrusion with in-line supercritical carbon. J. Appl. Polym. Sci. 103, 884–892 10.1002/app.25226

[B69] Vaia R. A. Ishii H. Giannelis E. P. (1993). Synthesis and properties of two dimensional nanostructures by direct intercalation of polymer melts in layered silicates. Chem. Mater. 5, 1694–1696 10.1021/cm00036a004

[B70] Venditto V. De Girolamo Del Mauro A. Mensitieri G. Milano G. Musto P. Rizzo P. (2006). Anisotropic guest diffusion in the δ crystalline host phase of syndiotactic polystyrene: transport kinetics in films with three different uniplanar orientations of the host phase. Chem. Mater. 18, 2205–2210 10.1021/cm051657s

[B71] Wang X. Jana S. C. (2013). Syndiotactic polystyrene aerogels containing multi–walled carbon nanotubes. Polymer 54, 750–759 10.1016/j.polymer.2012.12.025

[B72] Wang Y. Alhassan S. M. Yang V. H. Schiraldi D. A. (2013). Polyether-block-amide copolymer/clay films prepared via a freeze-drying method. Compos. Part B 45, 625–630 10.1016/j.compositesb.2012.05.017

[B73] Wei T. Y. Chen C. H. Chang K. H. Lu S. Y. Hu C. C. (2010). A Cost-effective supercapacitor material of ultrahigh specific capacitances: spinel nickel cobaltite aerogels from an epoxide-driven sol-gel process. Adv. Mater. 22, 347–351 10.1002/adma.200902175 20217716

